# Prevalence and factors associated with medication adherence in children with central precocious puberty: a cross-sectional study

**DOI:** 10.3389/fphar.2023.1269158

**Published:** 2024-01-08

**Authors:** Chunsong Yang, Xia Song, Jin Wu, Lingli Zhang

**Affiliations:** ^1^ Department of Pharmacy, West China Second University Hospital, Sichuan University, Chengdu, China; ^2^ Evidence-Based Pharmacy Center, West China Second University Hospital, Sichuan University, Chengdu, China; ^3^ NMPA Key Laboratory for Technical Research on Drug Products in Vitro and in Vivo Correlation, Chengdu, China; ^4^ Key Laboratory of Birth Defects and Related Diseases of Women and Children, Sichuan University, Ministry of Education, Chengdu, China; ^5^ West China School of Pharmacy, Sichuan University, Chengdu, China; ^6^ Department of Children’s Genetic Endocrinology and Metabolism, West China Second University Hospital, Sichuan University, Chengdu, China; ^7^ Chinese Evidence-Based Medicine Center, West China Hospital, Sichuan University, Chengdu, China

**Keywords:** central precocious puberty, medication adherence, influencing factors, crosssectional survey, children

## Abstract

**Objectives:** This study aimed to investigate the prevalence and influencing factors of medication adherence in children with central precocious puberty (CPP), and provide references for clinical practice.

**Methods:** Children under 12 years of age with CPP and their caregivers at a women’s and children’s hospital were selected for a cross-sectional study from February to May 2023, and a questionnaire was used to collect basic characteristics of pediatric patients and their caregivers, information of medication, cognition of medication, and medication adherence. The 8-item Morisky Medication Adherence Scale (MMAS-8) was adopted to assess medication adherence, and the potential influencing factors were explored by univariate and multivariate analyses.

**Results:** A total of 125 valid questionnaires were collected. The medication adherence rate of children with CPP reported by caregivers was 76.0%. The univariate analysis showed that the percentage of parental caregivers (*p* = 0.027), the age of caregivers (*p* = 0.029), the education level of caregivers (*p* = 0.001), the financial burden (*p* < 0.000), the incidence of adverse effects (*p* = 0.008), and the cognition of medication including the importance of medication (*p* = 0.002), the dosage of medication (*p* = 0.002), the adverse effects of medication (*p* = 0.007), the harm of non-compliance with medication (*p* < 0.000), and the evaluation of the price of medication (*p* = 0.003) in the poor adherence group were significantly inferior to those in the better adherence group. The multivariate analysis showed that the higher incidence of adverse effects (*p* = 0.20), not understanding the harm of non-compliance with medication (*p* = 0.004), and evaluation of the price of medication as expensive (*p* = 0.043) were independent risk factors for poor medication adherence.

**Conclusion:** Medication adherence in children with CPP is relatively better, and the factors leading to poor medication adherence are mainly on the caregivers of pediatric patients. It is recommended to increase the health education among caregivers of children with CPP to enhance the cognition of the condition and medication, and further improve the therapeutic efficacy for CPP.

## 1 Introduction

Central precocious puberty (CPP) is a common condition in which children experience rapid development of reproductive organs and secondary sexual characteristics presentation before 8 years old in girls and 9 years old in boys due to the early activation of the hypothalamic-pituitary-gonadal axis (HPGA) (Cheuiche et al., 2021). The incidence and prevalence of CPP vary among different races, but the onset of puberty is becoming earlier and the incidence of CPP is increasing year by year in various countries in the world (Bräuner et al., 2020). The premature epiphyseal closure due to rapid physical development may result in low height in adulthood, and the early onset of puberty and menarche in girls may also induce heavy mental burdens and serious psychosocial problems for patients and their families (Cheong et al., 2015). The recognized standard treatment regimen for CPP is gonadotropin-releasing hormone agonist (GnRHa), which aims to inhibit the overproduction of gonadotropin-releasing hormone (GnRH) secreted by the hypothalamus through regulation of HPGA, thereby reducing the synthesis and secretion of sex hormones and delaying the development of gonadal glands. However, the application of GnRHa for CPP may cause the body to experience a range of side effects that potentially affect the medication adherence of patients, including hot flashes, vaginal bleeding, and injection-site reactions (Cheuiche et al., 2021).

Medication adherence refers to the medication behaviors of the patient meeting the regimen agreed to with their healthcare provider (Malaeb et al., 2023). Previous studies have shown that medication adherence is associated with the therapeutic effect of medication (Chen et al., 2023; Özdemir et al., 2023). However, the COVID-19 pandemic has caused millions of people isolated at home for extended periods in recent years, which influenced the regular schedule of hospital visits and medication of CPP patients (Chen et al., 2022; Chioma et al., 2023). Currently, there is a lack of relevant studies on medication adherence and its influencing factors in children with CPP. Thus, this study aimed to investigate the current status of medication adherence in pediatric patients with CPP and comprehensively analyze its influencing factors, to provide references for the development of clinical interventions targeting better control of the condition and improvement of therapeutic efficacy among children.

## 2 Methods and material

### 2.1 Study design, setting and population

A cross-sectional study of medication adherence was conducted among children with CPP and their caregivers admitted to the pediatric outpatient clinic of West China Second University Hospital, Sichuan University between February and May 2023. The eligible participants were: 1) those who met the diagnostic criteria for CPP according to the Expert Consensus on Diagnosis and Treatment of Central Precocious Puberty (2022) (Subspecialty Group of Endocrinologic, Hereditary and Metabolic Diseases, the Society of Pediatrics, Chinese Medical Association, Editorial Board, Chinese Journal of Pediatrics, 2023) and received medication for more than 3 months; 2) the age of pediatric patients was less than 12 years old; and 3) the children and their caregivers possessed basic reading and writing skills, and expression ability. Those who did not volunteer to participate in the study were excluded.

### 2.2 Data collection

A self-designed questionnaire was anonymously used to collect the information, including the basic characteristics of pediatric patients and their caregivers, information on medication, cognition of medication, and medication adherence. The researchers explained the purpose and content of the study to caregivers of children with CPP who met the inclusion criteria and handed the questionnaire to the caregivers to fill in after obtaining the informed consent of children and their guardians.

### 2.3 Instrument

The medication adherence was assessed based on the modified version of the 8-item Morisky Medication Adherence Scale (MMAS-8) that is suitable for CPP (Morisky et al., 2008). The items were as follows: “Item 1. Do you sometimes forget to carry out CPP drug treatment for your children? Item 2. Over the past 2 weeks, were there any days when you did not carry out CPP drug treatment for your children? Item 3. Have you ever cut back or stopped carrying out CPP drug treatment for children without telling doctor because you felt worse when you took it? Item 4. When you travel or leave home, do you sometimes forget to bring along your CPP medicine during the treatment period? Item 5. Did you carry out CPP drug treatment last month or yesterday? Item 6. When you feel like your children’ disease is under control, do you sometimes stop carrying out CPP drug treatment? Item 7. Taking medication every month or everyday is a real inconvenience for some people. Do you ever feel hassled about sticking to CPP treatment plan for your children? Item 8. How often do you have difficulty remembering to carry out CPP drug treatment?” The instrument consists of eight items and scored according to the responses of participants, where the first 7 questions were answered with “yes” or “no”, with “yes” scored 0, “no” scored 1, and the third question was scored conversely. While the eighth question was scored 1, 0.75, 0.50, 0.25, and 0 for the answer “never”, “occasionally”, “sometimes”, “often”, and “always, respectively. The total score is the sum of the scores for each item ranging from 0–8. The higher the score, the better the medication adherence of the child. We set a total score of less than 6 as poor adherence and more than 6 as better adherence.

### 2.4 Data analysis

Statistical analyses were performed using SPSS 26.0 software. Quantitative data were expressed as mean and standard deviation (Mean ± SD), and qualitative data were as frequency and percentage (n, %). Normally distributed data were analyzed using the variance analysis and the non-normally distributed data were analyzed using the Mann-Whitney *U* test for quantitative data. The qualitative data were analyzed using the chi-squared test or Fisher’s exact test. The factors with *p* ≤ 0.05 in the univariate analysis were subjected to the multivariate logistic regression model to explore the potential factors associated with medication adherence. P ≤ 0.05 was considered to be statistically significant.

## 3 Results

### 3.1 Characteristics of participants

A total of 125 questionnaires were obtained from children with CPP and their caregivers in this study. Of the CPP patients included, girls were predominantly 84.8% (106/125), with a mean age of 9.90 ± 1.27 years and a mean duration of the condition of 1.45 ± 1.19 years. Among their caregivers, 93.6% (117/125) were parents, and the age of the caregivers was mainly 30–44 years old, accounting for 84.0% (105/125). 30.4% (38/125) patients were treated GnRHa combined with growth hormone (GH). The characteristics of pediatric patients and their caregivers are detailed in Table 1.

**TABLE 1:** The characteristics of including participants.

Variables	Total (n = 125)	Medication compliance		χ^2^/t	*P*
		Better (n = 95)	Poor (n = 30)		
**Pediatric patients**					
**Sex (n, %)**				1.281	0.258
Boys	19 (15.2)	12 (12.63)	7 (23.32)		
Girls	106 (84.8)	83 (87.37)	23 (76.67)		
**Age (years, mean ± SD)**	9.90 ± 1.28	9.98 ± 1.24	9.66 ± 1.37	1.203	0.231
**BMI (kg/m** ^**2**^ **, mean ± SD)**	18.28 ± 2.56	18.35 ± 2.63	18.03 ± 2.36	0.612	0.542
**Duration of** **condition** **(years, mean ± SD)**	1.45 ± 1.19	1.40 ± 1.25	1.60 ± 0.96	0.806	0.422
**Menarche or spermatorrhea (n, %)**				1.419	0.234
Yes	24 (19.2)	16 (16.84)	8 (26.67)		
No	101 (80.8)	79 (83.16)	22 (73.33)		
**Comorbidities (n, %)**				0.000	1.000
Yes	13 (10.4)	10 (10.53)	3 (10.00)		
No	112 (89.6)	85 (89.47)	27 (90.00)		
**Caregivers**					
**Role**				4.874	0.027*
Parent	117 (93.6)	92 (96.8)	25 (83.3)		
Non-parent	8 (6.4)	3 (3.2)	5 (16.7)		
**Age (n, %)**				7.108	0.029*
30–44 years old	105 (84.0)	82 (86.31)	23 (76.67)		
45–59 years old	12 (9.6)	10 (10.53)	2 (6.67)		
≥60 years old	8 (6.4)	3 (3.16)	5 (16.66)		
**Education level (n, %)**				13.838	0.001*
Senior high school and below	34 (27.2)	25 (26.32)	19 (63.33)		
Undergraduate	70 (56.0)	60 (63.16)	10 (33.33)		
Graduate and above	11 (8.8)	10 (10.52)	1 (3.34)		
**Place of residence (n, %)**				5.387	0.068
Urban	101 (80.8)	81 (85.26)	20 (66.67)		
Suburb	18 (14.4)	10 (10.53)	8 (26.67)		
Rural	6 (4.8)	4 (4.21)	2 (6.66)		
**Marital status (n, %)**				3.168	0.075
Single-parent	8 (6.4)	4 (4.21)	4 (13.33)		
Married	117 (93.6)	91 (95.79)	26 (86.67)		
**Financial burden (n, %)**				14.646	0.000*
Difficult to pay	27 (21.6)	13 (13.69)	14 (46.67)		
Able to pay	98 (78.4)	82 (86.31)	16 (53.33)		
**Information of medication**					
**Type of medication (n, %)**				0.001	0.972
GnRHa alone	104 (83.2)	80 (84.21)	26 (86.67)		
GnRHa combined GH	21 (16.8)	15 (15.79)	4 (13.33)		
**Duration of medication (n, %)**				2.413	0.491
<6 months	34 (27.2)	29 (30.53)	5 (16.67)		
6–12 months	42 (33.6)	30 (31.58)	12 (40.00)		
12–48 months	28 (22.4)	20 (21.05)	8 (26.66)		
≥48 months	21 (16.8)	16 (16.84)	5 (16.67)		
**Adverse effects (n, %)**				7.086	0.008*
Yes	13 (10.4)	6 (6.32)	7 (23.33)		
No	112 (89.6)	89 (93.68)	23 (76.67)		
**Cognition of medication**					
**Whether understanding the importance of medication (n, %)**				10.017	0.002*
Yes	115 (92.0)	92 (96.84)	23 (76.67)		
No	10 (8.0)	3 (3.16)	7 (23.33)		
**Whether understanding the dosage of medication (n, %)**				9.384	0.002*
Yes	117 (93.6)	93 (97.89)	24 (80.00)		
No	8 (6.4)	2 (2.11)	6 (20.00)		
**Whether understanding the adverse effects of medication (n, %)**				7.197	0.007*
Yes	60 (48.0)	52 (54.74)	8 (26.67)		
No	65 (52.0)	43 (45.26)	22 (73.33)		
**Whether understanding the harm of non-compliance with medication (n, %)**				14.458	0.000*
Yes	110 (88.0)	90 (94.74)	20 (66.67)		
No	15 (12.0)	5 (5.26)	10 (33.33)		
**Degree of satisfaction with the efficacy of treatment (n, %)**				2.602	0.107
Yes	49 (39.2)	41 (43.16)	8 (26.67)		
No	76 (60.8)	54 (56.84)	22 (73.33)		
**Evaluation of the medication regimen (n, %)**				1.526	0.217
Simple	111 (88.8)	82 (86.32)	29 (96.67)		
Complex	14 (11.0)	13 (13.68)	1 (3.33)		
**Evaluation of the price of medication (n, %)**				8.894	0.003*
Expensive	62 (49.6)	40 (42.11)	22 (73.33)		
Inexpensive	63 (50.4)	55 (57.89)	8 (26.67)		

### 3.2 Medication adherence

The mean score of MMAS-8 reported by caregivers was 6.95 ± 1.38.76.0% of children (95/125) had better medication adherence, and 24.0% of children (30/125) had poor medication adherence which was mainly manifested by the difficulty of adhering to the treatment plan and forgetting to use the medication.

#### 3.2.1 Univariate analysis

There were no statistical differences between the two groups in children in terms of sex, age, BMI, duration of condition, the occurrence of the menarche or spermatorrhea, comorbidities, type of medication, and duration of medication (*p* > 0.05). The incidence of adverse effects in the group with poor medication adherence was significantly higher than that in the group with better medication adherence (*p* = 0.008) (Table 1).

There were no statistical differences between the two groups in caregivers in terms of place of residence and parental marital status (*p* > 0.05). The age of caregivers (*p* = 0.029), the financial burden (*p* < 0.000), and evaluation of the price of medication as expensive (*p* = 0.003) were significantly higher in the poor medication adherence group than in the better medication adherence group, while the percentage of parental caregivers (*p* = 0.027), the education level of caregivers (*p* = 0.001), understanding the importance of medication (*p* = 0.002), understanding the dosage of medication (*p* = 0.002), understanding the adverse effects of medication (*p* = 0.007), and understanding the harm of non-compliance with medication (*p* = 0.000) were significantly lower in the poor medication adherence group than in the better medication adherence group.

#### 3.2.2 Multivariate analysis

The logistic regression analysis showed that medication adherence was poor in children with a higher incidence of adverse effects (*p* = 0.20), not understanding the harm of non-compliance with medication (*p* = 0.004), evaluation of the price of medication as expensive (*p* = 0.043), which may be independent risk factors for poor medication adherence. The result of multivariate analysis is shown in Table 2.

**TABLE 2:** The logistic regression analysis of factors influencing medication adherence.

Variables	β	SE	Wald	P	OR	95%CI
Constant	−1.121	2.079	0.291	0.590	0.326	
The role of caregivers	3.768	2.327	2.622	0.105	43.290	0.452–4,141.635
The age of caregivers	−1.552	1.074	2.088	0.148	0.212	0.026–1.739
The educational level of caregivers	−0.180	0.543	0.110	0.740	0.835	0.288–2.419
Financial burden	−0.645	0.642	1.010	0.315	0.525	0.149–1.846
Adverse effects	−2.052	0.885	5.374	0.020	0.128	0.023–0.728
Whether understanding the importance of medication	1.218	1.213	1.009	0.315	3.382	0.314–36.435
Whether understanding the dosage of medication	1.155	1.407	0.674	0.412	3.175	0.201–50.083
Whether understanding the adverse effects of medication	−0.223	0.598	0.139	0.709	0.800	0.248–2.584
Whether understanding the harm of non-compliance with medication	2.525	0.888	8.077	0.004	12.488	2.189–71.236
Evaluation of the price of medication	−1.495	0.740	4.086	0.043	0.224	0.053–0.956

## 4 Discussion

A total of 125 children with CPP and their caregivers were investigated in this study, and the overall level of medication adherence was relatively better. Our results showed that the independent risk factors affecting poor medication adherence in pediatric patients with CPP included the higher incidence of adverse effects, not understanding the harm of non-compliance with medication, and evaluation of the price of medication as expensive. This study provided a reference for medical personnel to understand the current situation of medication compliance among pediatric patients with CPP more comprehensively, and formulate more appropriate therapeutic regimens for patients.

Compared with the group with better medication adherence, the group with poor medication adherence had a higher incidence of adverse effects, such as depression and growth retardation, *etc.*, as reported by caregivers. Serious adverse effects in pediatric patients may cause anxiety among their caregivers who are worried about the harmful impact on their children’s normal life, thus reducing their confidence in adhering to the treatment and leading to poor adherence. In the absence of correct and timely intervention, children with CPP may have a greater psychological burden due to the lower adult height, and easily develop negative emotions affecting mental health, such as low self-esteem, anxiety, and depression (López-Miralles et al., 2022). Therefore, the treatment for CPP is still of great importance and the advantages outweigh the disadvantages. Medical personnel should help pediatric patients and their caregivers to establish confidence in overcoming the condition before treatment, and eliminate their concerns and worries during treatment through effective health education and psychological guidance.

Our results also found that caregivers in the poor medication adherence group had a worse cognition of the harm of non-compliance with medication, which is in agreement with Zhang et al.’s findings (Zhang et al., 2022). Caregivers with a lower cognition of medication adherence were more likely to present behaviors of difficulties in obeying the medication and had less confidence in treatment. Therefore, further strengthening the education of cognition of the condition and medication is still necessary, especially for caregivers with low education levels, to improve the level of control of the condition and effectively improve medication adherence in pediatric patients.

Some studies indicated that medication adherence was lower among those with financial difficulties and consideration of the medical expenses as high, which matched those observed in our study (Liu et al., 2022; Drouin et al., 2023). Most medications for CPP are the original brands with expensive prices in China, and our study showed that 49.6% of families considered that the medication was expensive and 21.6% of them had difficulty in paying for the medical expenses. Due to the characteristic of the long period of treatment and the administration of medication by injection, pediatric patients and their caregivers need to go to the hospital frequently to get prescriptions and injections, rendering it higher expenses than common diseases. Although most of the families had medical insurance or commercial insurance, the medication was not reimbursed or covered. The heavy financial burden further affected the medication adherence of patients and their families.

The GnRHa for CPP is a prescription medication with a fixed dosage and requires injection administration under the assistance of nurses. Most pediatric patients and their caregivers generally obey the therapeutic regimen and would not self-adjust the dosage if they felt the height improved or failed to improve, thus these groups have good medication adherence. In addition, children with CPP have a higher social acceptance and better social expectations than those with other chronic diseases or conditions (e.g., nervous and mental disorders), and their caregivers may prefer to report their children as exhibiting good behavior and high levels of adherence. As shown in the study reported by Goodfellow et al. (2015), parent-reported and child-reported medication adherence was the statistical difference. It should be noted to consider adding questionnaires filled by children to get more comprehensive information and compare it with parent-reported outcomes in the follow-up study on medication adherence of pediatric patients.

There is not yet a ‘gold standard’ measure for monitoring patient adherence, subjective measurement (i.e., Morisky scale) and objective measurement (therapeutic drug monitoring (TDM), electronic devices, and pickup/refill rates) were both widely used, and each method has its advantages and disadvantages (Hoegy et al., 2019; Davies et al., 2020). Although we did not used some objective measures to assess the medication adherence, many studies produced moderate to high correlation between both self-reported questionnaires (SRQs) and monitoring devices (Monnette et al., 2018).

This study also has some limitations. Firstly, the study was conducted only in the pediatric outpatient clinic of West China Second University Hospital, Sichuan University, and lacked data on children who had discontinued medication, which may not accurately reflect the overall characteristics of patients with CPP. Moreover, the caregivers may have recall bias regarding their children’s medication due to the long-term treatment, for which medication adherence may vary in different treatment stages. In addition, the influencing factors associated with medication adherence included in this study were relatively limited, and the questionnaire fillers were the caregivers of pediatric patients, without taking the children’s own beliefs and attitudes toward medication into account, so the results may not reflect the subjectivity of the children in the process of medication administration. Finally, we used the self-reporting of caregivers to assess medication adherence, the findings might therefore not reflect their actual practice, so it is necessary to use multiple methods assessment.

## 5 Conclusion

In conclusion, the current status of medication adherence in children with CPP is relatively better, and the higher incidence of adverse effects, the poor cognition of medication, and the expensive price of treatment are independent risk factors for poor medication adherence. Medical personnel should increase doctor-patient communication and strengthen health education, especially for caregivers with insufficient cognition of the condition, to enhance their confidence in the treatment and medical personnel. At the same time, the health insurance department of government should pay more attention to the financial burden of families with CPP patients, and formulate policies to reduce the burden and improve the accessibility of medicines.

## Data Availability

The raw data supporting the conclusions of this article will be made available by the authors, without undue reservation.
